# Reviewed article: Silva HRF, Júnior WPH, Magalhães KN, Magalhães DLN, Santos AQS, Ferreira MAD. Suicide attempts by poisoning recorded at a toxicological information and assistance center Fortaleza, Brazil, 2022: a cross-sectional study. Epidemiol Serv Saude. 2025:34;e20240885.

**DOI:** 10.1590/S2237-96222025v34e20240885.a

**Published:** 2025-08-11

**Authors:** Tayanny Margarida Menezes Almeida Biase

**Affiliations:** Universidade Estadual de Campinas, School Pharmaceutical Science

**Table:** 

Rating	Excellent	Good	Average	Below average	Poor
Interest		✓			
Quality			✓		
Originality		✓			
Overall		✓			

**Table:** 

Questionnaire	Yes	No	Not applicable
Does the manuscript contain new and significant information to justify publication?	✓		
Does the Abstract (Summary) clearly and accurately describe the content of the article?	✓		
Is the problem significant and concisely stated?	✓		
Are the methods described comprehensively?	✓		
Are the interpretations and conclusions justified by the results?	✓		
Is adequate reference made to other work in the field?	✓		

## Manuscript Structure

Length of article is: Adequate

Number of tables is: Adequate

Number of figures is: Adequate

Please state any conflict(s) of interest that you have in relation to the review of this paper (state “none” if this is not applicable): None

## Comments

O artigo aborda um tema relevante para a saúde pública, trazendo dados epidemiológicos para embasar políticas de prevenção ao suicídio e controle do acesso a substâncias tóxicas. O estudo está bem estruturado, com metodologia adequada ao delineamento proposto e uma discussão embasada na literatura científica.

Aqui estão algumas sugestões para a melhoria do artigo:

Resumo

O resumo sintetiza bem os achados do estudo, incluindo os principais resultados numéricos. No entanto, os métodos poderiam ser mais detalhados para esclarecer melhor a reprodutibilidade do estudo.

• Sugiro expandir ligeiramente a descrição dos métodos no resumo, especificando melhor a população estudada e a abordagem estatística.

• Verificar se todas as palavras-chave constam no DeCS/MeSH, garantindo padronização.

Introdução

A introdução contextualiza bem a importância do tema, citando dados epidemiológicos globais e nacionais sobre suicídio e intoxicações. O problema de pesquisa está bem delimitado, destacando a relevância da intoxicação como método de tentativa de suicídio e sua conexão com fatores sociais e psicológicos. O texto segue uma progressão lógica, partindo do contexto geral (suicídio global), para o nacional (Brasil), e depois para o foco específico (intoxicação como método).

• Algumas frases são longas e poderiam ser simplificadas para melhorar a fluidez. Exemplo:

Texto original: “O Brasil está entre os 10 países com o maior número de óbitos por essa causa. Conforme dados do Ministério da Saúde, ocorreu um total de 112.230 mortes por suicídio entre 2010 e 2019. Nesse período, o número anual de óbitos aumentou 43%, de 9.454 em 2010 para 13.523 em 2019.”

Sugestão: “O Brasil está entre os 10 países com mais óbitos por suicídio. Entre 2010 e 2019, o número de mortes aumentou 43%, passando de 9.454 para 13.523, segundo o Ministério da Saúde.”

• O texto poderia incluir mais referências internacionais recentes para contextualizar tendências globais no uso de intoxicação como método de tentativa de suicídio.

• As referências 5 e 6 são antigas, sugiro procurar mais atuais.

• Há uma repetição excessiva de conceitos sobre comportamento suicida. Em vez de reforçar a importância do tema em vários parágrafos, a introdução poderia ser mais concisa.

• Seria interessante uma breve menção ao contexto regional (Ceará) na introdução, pois o estudo foca nesse estado.

Métodos

• Por que o estudo analisou dados somente de janeiro a dezembro de 2022? Por que não incluiu anos anteriores?

• Não há menção sobre como foram tratados casos com informações incompletas.

• Não fica claro se houve exclusão de casos duplicados (por exemplo, pacientes que tentaram suicídio mais de uma vez no período e foram atendidos novamente).

• Variáveis: inserir referência sobre ATC e que foram reportados também por grupo terapêutico (já que a tabela 1 reporta os resultados dessa forma).

• Fontes de dados: descrever mais detalhes sobre o Sistema Brasileiro de Dados de Intoxicações dos Centros de Informação e Assistência Toxicológica, ele está vinculado a Anvisa? Como os dados estão organizados? Por ano, meses?

Resultados

• No primeiro parágrafo: “Os grupos de agentes tóxicos envolvidos com maior frequência nas tentativas de suicídio foram medicamentos (n=232; 64,4%), praguicidas (n=68; 18,9%), saneantes domissanitários (n=0; 5,6%), drogas de abuso (n=1; 0,3%) e agentes que não foram identificados (n=2; 0,6%)”. Saneantes domissanitários tem o n = 0 e porcentagem de 5,6%. Acredito que algum dado foi excluído da frase, pois não condiz com a tabela 1.

• No segundo parágrafo: “Ressalta-se que, embora tenham ocorrido 232 casos, foram utilizados 505 medicamentos, pois um único indivíduo pode utilizar dois ou mais medicamentos”. Isso deve ser detalhado na sessão de métodos sobre a coleta das variáveis.

• O excesso de números e percentuais pode dificultar a leitura. Em alguns trechos, os dados poderiam ser agregados em frases mais fluidas ao invés de listar valores numéricos. Por exemplo:

Texto original: “Dos 360 casos de tentativa de suicídio, 232 (64,4%) envolveram medicamentos, 68 (18,9%) praguicidas e 20 (5,6%) saneantes domissanitários.”

Sugestão de reescrita: “A maioria das tentativas de suicídio envolveu medicamentos (64,4%), seguidos por praguicidas (18,9%) e saneantes domissanitários (5,6%).”

• Alguns trechos apresentam frases longas e complexas, o que pode dificultar a compreensão. Exemplo:

Texto original: “A exposição aos saneantes domissanitários foi mais frequente entre indivíduos do sexo feminino (10 casos, 50,0%) e na faixa etária de 20-29 anos (3 casos, 15,0%).”

Sugestão de reescrita: “Os saneantes domissanitários foram mais usados por mulheres (50%) e por indivíduos de 20 a 29 anos (15%).”

• Alguns resultados poderiam ser discutidos mais brevemente na seção de resultados, deixando interpretações detalhadas para a discussão.

Discussão

• A discussão sintetiza bem os principais achados, destacando que a maioria das tentativas de suicídio por intoxicação envolveu medicamentos, seguidos por praguicidas e saneantes domissanitários.

• Alguns resultados são repetidos de forma muito semelhante à seção de resultados, sem adicionar interpretação ou implicações mais amplas.

• Poderia haver mais ênfase nas razões por trás dos achados, por exemplo, por que os medicamentos foram os agentes tóxicos mais utilizados?

• A comparação com outros estudos poderia ser mais crítica. O artigo menciona que os resultados são semelhantes a outras pesquisas, mas não discute diferenças regionais ou contextuais que poderiam influenciar os achados.

• Não há exploração suficiente de possíveis explicações para divergências com a literatura.

• A questão do acesso aos medicamentos poderia ser melhor explorada (exemplo: regulação da venda de psicotrópicos, campanhas de conscientização sobre armazenamento seguro).

• O papel da saúde mental e do suporte psicossocial poderia ser melhor relacionado aos achados.

• Algumas limitações importantes podem ser exploradas:

Possível subnotificação de casos (por exemplo, tentativas de suicídio não atendidas pelo centro de toxicologia).

Ausência de dados clínicos e psicossociais detalhados dos pacientes, o que poderia enriquecer a análise.

Viés de informação, já que os dados foram coletados retrospectivamente e podem conter erros de registro.

• Reforçar na conclusão o impacto dos achados para a formulação de estratégias preventivas.

Tabelas

• Como a amostra é relativamente pequena (360 casos), os valores absolutos já são suficientemente informativos.

• Percentuais poderiam ser mantidos apenas para categorias-chave, como distribuição por sexo, principais grupos de agentes tóxicos e taxas de letalidade.

• Algumas tabelas listam muitas subdivisões de medicamentos e praguicidas, o que pode ser resumido em categorias maiores com detalhes no texto.

• Por exemplo, ao invés de listar todas as classes de medicamentos, poderia agrupar como:

Psicofármacos (antidepressivos, ansiolíticos, antipsicóticos)

Outros medicamentos (analgésicos, anti-inflamatórios etc.)

• Em vez de uma única tabela extensa, poderia-se dividir em tabelas menores para facilitar a leitura. Exemplo:

Tabela 1: Perfil demográfico (idade, sexo, ocupação).

Tabela 2: Distribuição dos agentes tóxicos utilizados.

Tabela 3: Desfecho clínico e necessidade de internação. 

